# Estimated relative potential for airborne SARS-CoV-2 transmission in a day care centre

**DOI:** 10.1016/j.heliyon.2024.e30724

**Published:** 2024-05-06

**Authors:** Ilpo Kulmala, Aimo Taipale, Enni Sanmark, Natalia Lastovets, Piia Sormunen, Pekka Nuorti, Sampo Saari, Anni Luoto, Arto Säämänen

**Affiliations:** a VTT Smart Energy and Built Environment, Visiokatu 4, PO Box 1300, FI-33101, Tampere, Finland; b Helsinki University Hospital, Department of Otorhinolaryngology and Phoniatrics – Head and Neck Surgery, Helsinki, Finland; c University of Helsinki, Helsinki, Finland; d Tampere University, Faculty of Built Environment, Civil Engineering Unit, Korkeakoulunkatu 5D, FI-33720, Tampere, Finland; e Tampere University, Faculty of Social Sciences, Health Sciences Unit, Arvo Ylpön Katu 34, 33520, Tampere, Finland; f Tampere University of Applied Sciences, Kuntokatu 3, 33520, Tampere, Finland; g Granlund Oy, Malminkaari 21, 00700, Helsinki, Finland

**Keywords:** SARS-CoV-2, Infection probability, Modelling, Day care centre

## Abstract

We estimated the hourly probability of airborne severe acute respiratory coronavirus 2 (SARS-CoV-2) transmission and further the estimated number of persons at transmission risk in a day care centre by calculating the inhaled dose for airborne pathogens based on their concentration, exposure time and activity. Information about the occupancy and activity of the rooms was collected from day care centre personnel and building characteristics were obtained from the design values. The generation rate of pathogens was calculated as a product of viral load of the respiratory fluids and the emission of the exhaled airborne particles, considering the prevalence of the disease and the activity of the individuals. A well-mixed model was used in the estimation of the concentration of pathogens in the air. The Wells-Riley model was used for infection probability. The approach presented in this study was utilised in the identification of hot spots and critical events in the day care centre. Large variation in the infection probabilities and estimated number of persons at transmission risk was observed when modelling a normal day at the centre. The estimated hourly infection probabilities between the worst hour in the worst room and the best hour in the best room varied in the ratio of 100:1. Similarly, the number of persons at transmission risk between the worst and best cases varied in the ratio 1000:1. Although there are uncertainties in the input values affecting the absolute risk estimates the model proved to be useful in ranking and identifying the hot spots and events in the building and implementing effective control measures.

## Highlights

• A relatively simple but scientifically sound model for transmission of airborne infectious pathogens is presented.
• This model has been applied to a real-life building and activities in a day care centre for the transmission of SARS-CoV-2.
• Significant differences in infection probabilities and number of persons at risk between spaces and events were revealed.
• Ranking of high-risk rooms and activities enables an efficient focus on the risk mitigation measures.

## 1 Introduction

Infectious respiratory diseases are usually spread through direct contact, droplet, or aerosol transmission. The relative importance of each mode varies depending on the disease and it is often difficult to determine precisely. A case in point is severe acute respiratory coronavirus (SARS-CoV-2), which was initially believed to spread mainly through contact or droplets, but which is now increasingly recognized as also being transmitted by airborne route [[1], [2], [3]]. Consequently, there has been increasing interest in modelling the airborne dispersal of SARS-CoV-2 and the airborne transmission risk to help assess the effectiveness of various control measures [[4], [5], [6]].

When infected individuals sneeze, cough, sing, talk or even breathe, they emit particles over a wide size range, from submicron aerosols to droplets over 1 mm in size [7]. These particles may contain pathogens, the number of which depends on the pathogen concentration in the respiratory fluid and the volume of the droplet. Small droplets measuring <15 μm evaporate in fractions of seconds, leaving residual aerosol particles that can remain airborne for hours and if inhaled, can penetrate deep into the lungs. Larger droplets with a diameter of >100 μm evaporate too but at a much slower rate than the small droplets. The large droplets behave like ballistic projectiles and settle rapidly close to the point of emission. They are not inhalable but can cause droplet transmission risk when droplet spray lands on the facial mucous membranes of a susceptible person within close range. Aerosols of intermediate size – up to 100 μm – can be inhaled but are trapped in the upper respiratory tract.

Nicas and his fellow researchers [8] presented an airborne infection risk model that estimates the inhaled dose by taking into account the pathogen generation and removal mechanisms, and the alveolar deposition fraction of particles of different sizes. Later on, several SARS-CoV-2 airborne transmission risk assessment tools were developed based on the assumption that the pathogen emission rate is proportional to the viral load in expelled respiratory fluids and respiratory droplet generation rate [4,9,10]. These models need input parameters such as size distribution and emission rate of respiratory droplets. Jones et al. [11] reduced the uncertainties related to the probability of infection by calculating a relative exposure index based on the median number of deposited Ribonucleic acid (RNA) copies from inhalation and comparing the dose to a reference scenario.

Simpler versions of infection risk models are based on the well-known Wells-Riley ‘quanta’ concept [5,12]. In the context of SARS-CoV-2, a quantum is defined as the dose of airborne pathogens required to cause infection in 63 % of susceptible people. The quanta is typically estimated from epidemiological data after an outbreak investigation, using a reverse approach predicting the environmental conditions at the time of the outbreak to get infective quanta in the room (#/m^3^). Another way to estimate the quanta emission rate is to multiply the respiratory droplet emission rate with the viral load (ribonucleic acid (RNA) copies/mL) and the quanta-response relationship, which differs for different strains [6]. The quanta model relies on some assumptions, such as the concentration of quanta in the air not changing with time, quanta particles having a fixed but undefined size, and that an infected person emits quanta at a constant rate.

While useful for describing the effects of various factors on the transmission risk, most of the current models assume that there is at least one infected person emitting pathogens in the considered indoor environment. This may lead to unrealistically high risk predictions, especially in cases where there are only a few people present in the same indoor space at the same time. To get more realistic results, Li and Tang [13] presented a model where the number of infectors in indoor environments is related to the proportion of infectious people in the community for a certain time.

The developed models have been used to estimate the SARS-CoV-2 transmission risk in various indoor environments. Park and Kim [14] studied transmission risk on public transport and optimal seating arrangements. Li and Tang [13] examined the time-dependent risk in an outpatient building in Shenzhen, China, and found large variations in risk. Lelieveld et al. [4] conducted a modelling study to assess the risk of airborne transmission of SARS-CoV-2 in indoor example environments. They considered different premises, including an office, a classroom, a room where choir practice was taking place, and a reception space. However, few studies have focused on day care centres, although preschool settings are important in the spreading of infectious diseases in the community. In addition, most of the exhaled particle emission rate studies have been conducted with adult volunteers. Only few studies have investigated and compared emission rates for both adults and children, presenting contradictory results about the differences in emission rates between groups [15,16]. Age of children has not contributed to the emission rate [17].

In situations where the ventilation air flow rates are not known, as it usually happens in naturally ventilated buildings, the exposure to airborne pathogens emitted by infectious persons can be estimated from measured indoor carbon dioxide concentrations and assuming constant breathing and CO_2_ emission rates as presented by Rudnick and Milton [18]. Their model is simplified by ignoring pathogen deposition and inactivation. The increase in the indoor carbon dioxide concentration is proportional to air exhaled from other occupants in the same room and is assumed to be proportional to the probability of infection. However, the CO_2_ concentration may not be an accurate predictor of transmission risk because the CO_2_ emissions and viral particle emissions are not directly proportional to each other. Moreover, CO_2_ is removed by ventilation only while pathogens are removed also processes like inactivation and deposition [19].

Although all the models predict that an increased non-infectious air flow rate will reduce infection transmission, there is a lack of high-quality evidence of the effect of enhanced ventilation and air cleaning on transmission [20].

This study examined the potential for airborne SARS-CoV-2 transmission in a day care centre in Helsinki, Finland. The approach used in this work considers prevalence of the infection, activity, the number of the emitting and susceptible subjects in a room, the room volume, and the air exchange rate of the room. The concentration of SARS-CoV-2 virus in the room and the estimated number of persons at risk of contagion was calculated for each room in the day care building on an hourly basis, giving detailed spatiotemporal information on exposure in the day care centre.

## 2 Estimation of the potential for airborne transmission and estimation of persons at risk

The probability of airborne transmission was estimated in four steps. Firstly, the release of infectious aerosol was estimated in each room using the occupancy and activity information collected. Then the airborne concentration of pathogen-containing aerosols in different locations of the day care centre was calculated on an hourly basis for opening hours of the centre. Based on the concentration, the accumulated dose by inhalation was estimated using the activity and occupancy data. The probability of infection was then calculated using the exponential model first presented by Wells [21].

### 2.1 Release of pathogen-containing aerosol

Even though viral particles can be shed when sneezing and coughing, it is likely that sick individuals will stay at home when observable symptoms appear. Therefore, we assume that the emission of pathogen-laden droplets unknowingly occurs by asymptomatic or pre-symptomatic infectors during normal activities like speaking and singing. The focus is on the airborne droplet nuclei which are formed when water evaporates from the emitted respiratory droplets.

The resulting aerodynamic diameter of the expelled droplets depends on the non-volatile contents of the droplet composition and the ambient relative humidity, and has been estimated to be around 30 % of the original diameter in typical interiors [22]. Thus, under normal indoor conditions, droplets with an initial size of <15 μm dry within a fraction of a second to aerosols measuring <5 μm, which can reach the deeper parts of the respiratory tract when inhaled. Following Nicas et al. [8], the number of pathogens carried on particles was determined assuming that the distribution of viruses in droplets of different sizes is uniform.

Assuming that the viral load in expelled droplets is the same as in respiratory fluids, the viral aerosol emission rate *G* (RNA copies/s) of an infected person can be calculated by Equation (1):

(1) $$G=c_{v}\;q\cdot 10^{-9}$$

where *c*_*v*_ is the viral load in respiratory fluids (RNA copies/mL) and q (ng/s) is the mass emission rate of droplets with a diameter of less than 15 μm. The multiplier 10^−9^ comes from the conversion of ng to g assuming a density of 1 g/mL for the expelled droplets.

In principle, the pathogen emission rates can thus be estimated from directly quantifiable parameters. The viral concentration in respiratory fluids of those infected with SARS-CoV-2 varies largely between individuals and during the course of the disease [23,24]. The typical range in specimens from the throat and sputum of COVID-19 patients is 10^4^–10^11^ RNA copies/mL [4]. A median concentration of 10^6^ RNA copies/mL has been reported in studies by Yang et al. [25]. SARS-CoV-2 viral loads in infected children have been found to be similar to those in adults [26]. It is likely, however, that the disease is not spread evenly: studies of COVID-19 suggest that between 5 and 10 % of infected individuals are responsible for 80 % of secondary infections [27]. Based on the distributions it can be estimated that the saliva concentration of these active spreaders is about 5·10^8^ viral copies/mL. It can be argued that in the calculations it is therefore justified to use this value for emission estimations.

Alsved et al. [28] measured exhaled particle emission rates during different activities. Back-calculating to the mouth conditions, the median mass emission rates were 2 ng/s for breathing, 6 ng/s for talking and 12 ng/s for loud talking. Orton et al. [29] measured the respiratory particle emission rates at rest and while speaking or exercising, and found average mass emission rates of dried particles ranging from 0.003 to 0.2 ng/s, corresponding to 0.1 ng/s for resting, 3 ng/s for speaking and 6 ng/s for exercise at the mouth exit. Their measurements reported particles smaller than 10 μm, and it is likely that the volatile part of these droplets was evaporated before being detected by the measurement device. As the mass of spherical particles is proportional to the third power of the diameter, it may be assumed that the emission rates of droplets at the mouth exit were roughly 30 times higher than reported. In this study the activity in the day care centre was divided into three levels, with 1 corresponding to sleeping, 2 to normal activity and 3 to moderate exercise. The respective aerosol emission rates were assumed to be 2, 6 and 12 ng/s, which are in line with the modelled emission rate values by Ref. [30]. An order of magnitude estimation indicates that with saliva viral load ranging from 10^6^ to 10^8^ RNA copies/mL the viral shedding rate can vary between 10 and 4000 RNA copies/h.

### 2.2 The airborne concentration of pathogens

Once released in the indoor air, the number of infectious viruses C(t) (RNA copies/m^3^) is reduced by ventilation, inactivation, or deposition onto surfaces as expressed by Equation (2):

(2) $$\frac{dC\left(t\right)}{dt}=\frac{pNG}{V}-\;\lambda C\left(t\right)-\lambda_{IA}C\left(t\right)-\lambda_{D}C\left(t\right)-\lambda_{AC}C\left(t\right)$$

where p is the prevalence of the disease, *N* is the number of occupants in the space and *G* is the microbe generation rate of an infector (RNA copies/s). *V* is the volume of the space (m^3^). There are also factors that decrease the airborne concentration: ventilation air change rate *λ* (1/s), pathogen inactivation rate *λ*_*IA*_ (1/s) and deposition rate *λ*_*D*_ (1/s). There may also be air cleaners with an airflow rate of *q*_*AC*_ (m^3^/s) and filtration efficiency *E* to dilute airborne concentrations and *λ*_*AC*_ (1/s) is the clean air production rate of any air purifier, which equals the clean air delivery rate (CADR) divided by the room volume V.

Relative humidity and temperature are known to influence the inactivation of viruses [31,32] and this should be considered when choosing the inactivation rate term. Normally, temperature in the room is kept stable within comfortable limits. The relative humidity of the room may vary depending on the season. However, when comparing the situation of the rooms within the same building the relative humidity can be assumed to be constant.

Resuspension of settled pathogenic viruses from floors may also increase indoor air pathogen concentrations. However, the resuspension factor depends on the type of the flooring material. Yang et al. [33] found that resuspension emission factor from hard wood floorings similar to those in the day care centre ranged from 3 x10^−5^ - 8x10^−5^ due to human walking. Because of this relatively small fraction and on the other hand daily cleaning the effect of resuspension was neglected.

The resulting airborne pathogen concentration (RNA copies/m^3^) in a steady-state situation is obtained from Equation (3):

(3) $$C_{S}=\frac{pNG}{\left(\lambda +\lambda_{IA\;}+\lambda_{D\;}+\lambda_{AC}\right)V}=\frac{pNG}{\lambda_{TOT}V}$$

The pathogen concentration C(*t*) in a certain space varies according to the generation rate, which in turn depends statistically on the number of occupants and their activity. Assuming fully mixed conditions, the concentration in the room after a stepwise change in the generation rate can be expressed by Equation (4):

(4) $$C\left(t\right)=C_{S}\left(t_{i}\right)\exp\left(-\lambda_{TOT}\Delta t\right)+C_{s}\left(t_{i+1}\right)\left(1-\exp\left(-\lambda_{TOT}\Delta t\right)\right)$$

Where

(5) $$\Delta t=t-\;t_{i},t_{i}<t<\;t_{i+1\;}$$

where *C*_*S*_ *(t*_*i*_*)* refers to steady state concentration at *t*_*i*_ and *Δt* is time from stepwise change in the generation rate. In this study we used hourly based calculations, i.e. *t*_*i+1*_*–t*_*i*_ = 1 h in Equation (5). The initial concentration in each room in the morning before any activities is supposed to be zero.

### 2.3 Estimation of the dose

The dose a susceptible person gets when being in the same space with an infector is

(6) $$D=C_{ave}Btf_{i}$$

Where *C*_*ave*_ is the time-averaged concentration during time *t (RNA copies/m*^*3*^*), B* is breathing rate (m^3^/h), *t* is exposure time *(h)*, and *f*_*i*_ is the deposition factor of inhaled aerosols in the respiratory system. In the estimation of breathing rates, we utilised values indicated in the U.S. Environmental Protection Agency's (EPA) Exposure Factors Handbook (Table 1) [34]. In calculations we used the weighted values for breathing rate considering the number of adults and children as well as the reported activity in the room. As we are interested in airborne particles with a diameter of <5 μm which can be inhaled and deposited deep in the respiratory system, the deposition of inhaled particles in the respiratory tract must be considered. The overall deposition factor of 0.3 was used in these calculations corresponding the average deposition of particles measuring 1–5 μm in the tracheo-bronchial and alveolar regions [35,36].

**Table 1:** Inhalation rates for different activities in different age groups [34].

Activity	Children 1–3 years, Mean (95th percentile)	Children 3–6 years, Mean (95th percentile)	Adults 21–61, average, Mean (95th percentile)
Sleep/sedentary/passive	0.28 m^3^/h (0.39 m^3^/h)	0.27 m^3^/h (0.35 m^3^/h)	0.28 m^3^/h (0.41 m^3^/h)
Light	0.72 m^3^/h (0.96 m^3^/h)	0.66 m^3^/h (0.84 m^3^/h)	0.72 m^3^/h (0.96 m^3^/h)
Moderate	1.26 m^3^/h (1.74 m^3^/h)	1.26 m^3^/h (1.62 m^3^/h)	1.62 m^3^/h (2.28 m^3^/h)

Breathing rate used in equation (6) is calculated using weighted average for the number of children and adults in the room. This means that the susceptible dose is estimated for group of persons and the dose is not calculated for children nor adults separately.

### 2.4 Estimation of infection probability and number of persons at risk

Several statistical models based on the empirical data have been proposed to describe dose-response data [37]. The key feature of these models is that the risk of infection is proportional to the inhaled dose. A commonly used model is the exponential model first presented by Ref. [21]:

(7) $$P_{\mathrm{INF}}=\left(1-\exp\;\left(-0.693\frac{D}{D_{50}}\right)\right)$$

In Equation (7), *D* is the dose calculated by equation (6) and *D*_*50*_ is the dose causing infection on average to half of the exposed population. The average number of viral particles needed to initiate a COVID-19 infection is not known accurately, but given how rapidly the disease spread, it is likely to be relatively low. The reported values of infective dose vary largely, ranging from 100 to 14000 RNA copies, depending on the variant type [4,6]. In this study we utilise the approach by Lelieveld et al. [4] as they used the value of about 320 RNA copies.

It is interesting to examine how the occupancy pattern significantly affects the airborne infection risk and the expected number of infected people. When the inhaled dose is much smaller than the infective dose so that *D/D*_*50*_ ≪ 1, the infection risk is approximately proportional to the inhaled dose as shown by Equation (8):

(8) $$P_{\mathrm{INF}}=\left(1-\exp\;\left(-0.693\frac{D}{D_{50}}\right)\right)\;∼\;0.693\frac{D}{D_{50}}$$

and the statistically expected number of people at risk (E) is

(9) $$E=P_{\mathrm{INF}}\;N∼\frac{N^{2}G\cdot t\cdot Br\cdot f_{i}}{\;\lambda_{TOT}V}$$

Equation (9) reveals that the expected number of persons at risk (*E*) in a room where there are *N* persons is the product of infection probability and the number of persons so that the statistically expected number of persons who will get infected is proportional to the square of the number of occupants. This is the case when the other parameters, such as ventilation rate, remain constant. Limiting the number of occupants is thus one mitigation measure that can be effectively used to reduce the spread of the disease. This is supported by findings that COVID-19 shows high attack rates in indoor clusters, which suggests that the number of occupants is a key factor in getting infected [38].

## 3 Materials and methods

The studied day care centre is located in Helsinki, Finland, about 10 km north-east of the city centre. There are seven pre-school children's groups in the day care centre: four groups for 3–7-year-old children and three groups of younger 2–3-year-olds. The total number of children varied between 100 and 107 and the number of personnel was 17. The opening hours of the day care centre is from Monday to Friday between 6:15 a.m. and 5:30 p.m. The study was approved by ethical committee of Helsinki and Uusimaa (HUS/14231/2022) and was conducted according to Declaration of Helsinki.

### 3.1 Day care centre building

**Fig. 1:**
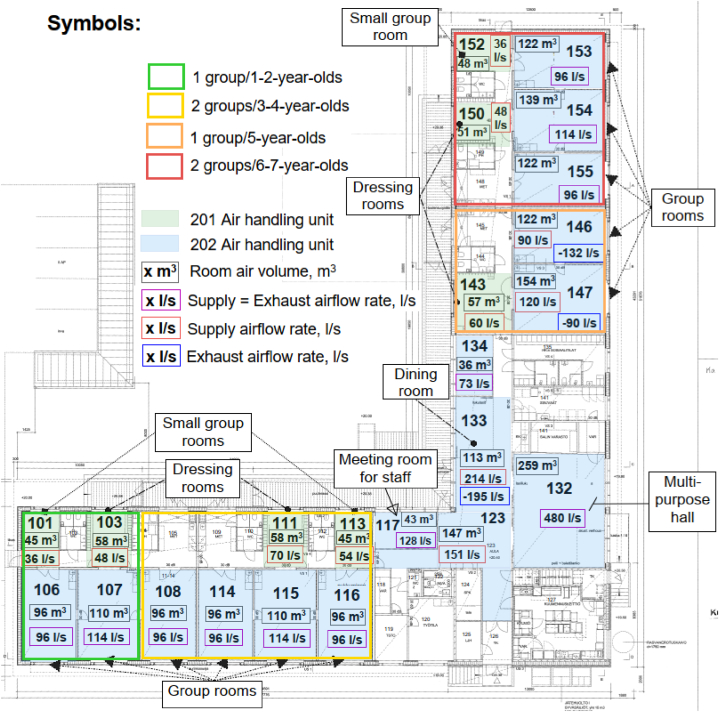
Spaces of interest in the day care centre, air volumes and design airflow rates.

The day care centre was built in 2013 in a suburban area of Helsinki. The L-shape building has an area of 1128 m^2^, of which living area for the day care operations is about 975 m^2^. The floor plan of the day care centre and the spaces of interest are shown in Fig. 1. The building was divided into four units where the day care groups spent most of the time indoors and in common spaces. Each unit consisted of several rooms such as group rooms, dressing rooms, small group rooms and toilets. In one unit there were 1–2 day care groups. Three groups of younger children (age 2–3) used group rooms 106, 107, 108, 114, 115 and 116 (Fig. 1). The other four groups were in rooms 146, 147, 153, 154 and 155. Children arrive in the building through four dressing rooms (room numbers 103, 111, 143 and 150) where they take off or put on their outer garments when coming in or going outside. Lunch is mainly served in rooms 133 and 134 and indoor physical exercise is performed in multi-purpose hall 132. Only those spaces where children and their nurses were on a regular basis were considered in calculations.

### 3.2 Ventilation system

**Fig. 2:**
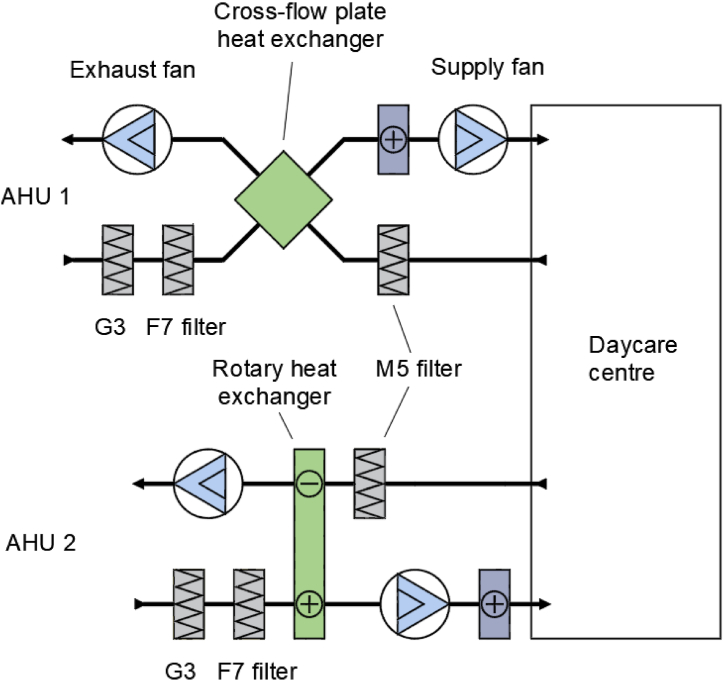
Schematics of air handling units 1 and 2.

The building has mechanical supply and exhaust ventilation systems. The spaces of interest are served by two air handling units (AHU): AHU 1 (constant air flow rate 3060 m^3^/h) and AHU 2 (constant air flow rate 7740 m^3^/h). The air handling units are equipped with filtration and with cross-flow plate or thermal wheel heat recovery systems (Fig. 2). Exhaust airlines have an air filter (M5) before the heat recovery system. Neither AHU used recirculation air. Direct drive fans are equipped with electronically commutated (EC) motors or frequency converters. The filters of the supply air units are disposable with the filtration class G3+F7. The properties of the air handling units are presented in Table 2 and Fig. 2.

**Table 2:** Characteristics of air handling units.

AHU	Supply line				Exhaust line			Heat recovery type
	Fan		Filter class	Heater power kW	Fan		Filter class	
	airflow rate, dm^3^/s	pressure Pa			airflow rate, dm^3^/s	pressure Pa		
1	810	300	G3 and F7	40	850	300	M5	Cross-flow
2	2130	300	G3 and F7	35	2150	300	M5	Rotary

Both air handling units operate during working days (Mon–Fri) from 4:00 to 18:00. The ventilation system in most rooms is constant air flow, but in the small group rooms, the dining room and multipurpose hall, the ventilation airflow can be manually increased. The design airflow rates correspond to the building regulations applied in Finland [39] and are shown in Fig. 1.

### 3.3 Occupancy and activities during day

**Fig. 3:**
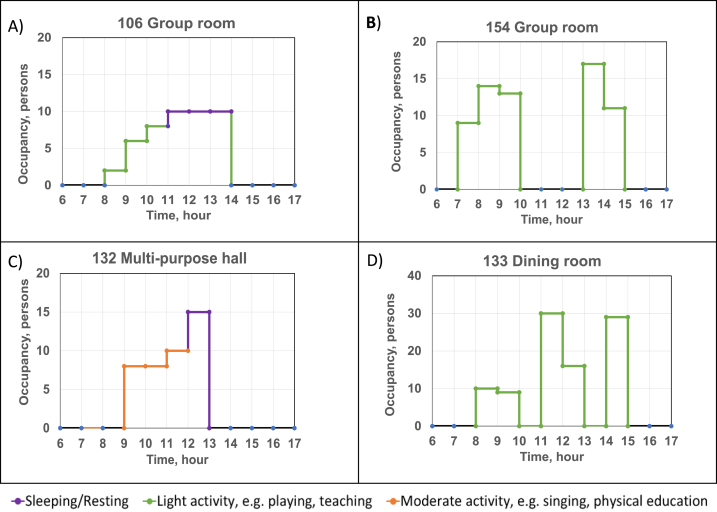
Examples of typical occupancy and activity profiles.

The hourly activities and number of occupants in different rooms were collected through a survey from the personnel of the day care centre. This information forms the occupancy and activity profile of the rooms used in the calculations. Several examples of these profiles are depicted in Fig. 3A–D. The groups of children spent time mainly in their own group rooms. Children from the younger age groups (Fig. 3A) spent 3 h resting and sleeping in rooms 106, 108, 114 and 116. Older groups (Fig. 3B) were located in rooms 146, 147, 153, 154 and 155 and had higher occupancy. The children in the older groups either had a short 1-h sleeping or resting time. The highest activity was observed in the multi-purpose hall (Fig. 3C). Breakfast and lunch as well as afternoon snack were served in dining room (Fig. 3D).

**Fig. 4:**
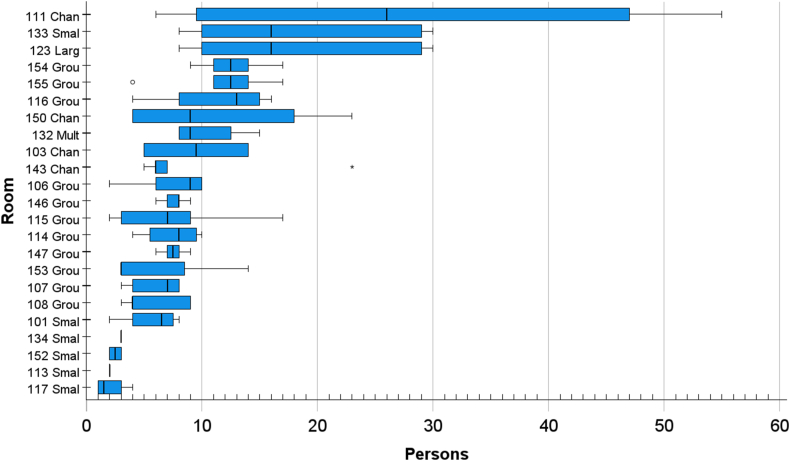
Boxplot showing hourly mean number of persons in rooms while occupied. The boundaries of the box are Tukey's hinges. The median is identified by a line inside the box. The length of the box is the interquartile range (IQR) computed from Tukey's hinges [40]. Values greater than three IQRs from the end of a box are labelled as extreme, and denoted with an asterisk (*). Values greater than 1.5 IQRs but less than 3 IQRs from the end of the box are labelled as outliers (o) (IBM SPSS Statistics 28.0.1).

**Fig. 5:**
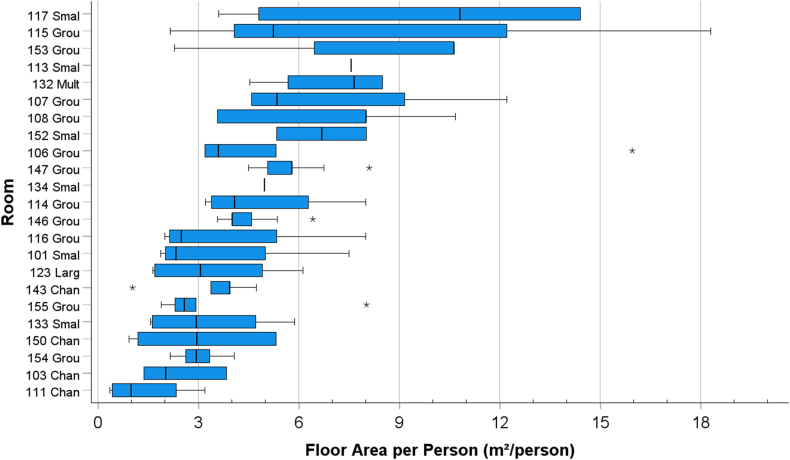
Boxplot of the hourly variation floor area per person in rooms. The boundaries of the box are Tukey's hinges. The median is identified by a line inside the box. The length of the box is the interquartile range (IQR) computed from Tukey's hinges [40]. Values greater than three IQRs from the end of a box are labelled as extreme, denoted with an asterisk (*). Values greater than 1.5 IQRs but less than 3 IQRs from the end of the box are labelled as outliers (o) (IBM SPSS Statistics 28.0.1).

The occupancy in the rooms varied during the day depending on their function. Sometimes the spaces were congested, e.g. changing rooms during arrival or departure, and dining rooms during lunch hour, whereas e.g. small group rooms were occupied by only a few persons. On the contrary, there were times when the spaces were unoccupied. Fig. 4 shows the variation in the number of persons in the room while they were occupied. The variation in the floor area per person is shown in Fig. 5. Sometimes the floor area per person was less than 3 m^2^/person.

### 3.4 Calculation of the transmission potential and the probability of infection in a day care centre

When the transmission potential and the probability of the infection was calculated, the underlying assumptions were adopted.

1. The pathogens and the pathogen emission rate are proportional to the droplet emission mass rate and viral concentration of the respiratory fluid.
2. Expelled droplets with an original diameter of <15 μm dry to 30 % of their initial size almost instantly, within fractions of a second. The resulting aerosols are smaller than 5 μm in size which move with the air currents and can carry pathogens.
3. The aerosol concentration in a room is uniform, meaning that the conditions are well-mixed.
4. The probability of infection transmission is proportional to the actual integer number of pathogens inhaled and deposited which follows a Poisson probability distribution.
5. The number of infectors in an indoor environment is proportional to the disease occurrence in the community.

The modelling includes several assumptions that drastically affect the calculated infection risk. First, the generation is the product of viral concentration in expiratory fluids and the emission rate, both of which have large variations. This increases uncertainties in the absolute values of infection transmission risk but does not affect the relative risks.

The infection transmission risk was calculated for each room in the day care centre using the collected hourly occupancy and building profiles. The prevalence of the disease was estimated based on the highest peak of prevalence [32] and multiplied by three to consider the non-reported cases. The actual number of COVID-19 cases compared to officially reported ones has been estimated to be 2.3 in a US study [41] and 4.5 in a German study [42].

A summary of the values used in the calculation of infection risks is presented in Table 3.

**Table 3:** Summary of the parameter values used in the calculations.

Parameter	Value	Unit	Reference
Prevalence of disease p	3 In sensitivity analysis 0.5–5	%	[[41], [42], [43]]
Number of persons in each space, N	1–55	–	Questionnaire
Exposure time	1	h	Questionnaire
Room volume V	33–340	m^3^	Construction drawings
Total air change rate λ_TOT_	0.9–3.8	1/h	Design values
Aerosol generation rate: resting, q	2	ng/s	[7,28]
Aerosol generation rate: moderate exercise, q	6	ng/s	
Aerosol generation rate: heavy exercise, q	12	ng/s	
Breathing rate B: children	0.27–1.26	m^3^/h	[34]
Breathing rate B: adults	0.28–1.62	m^3^/h	[34]
Viral load, c_v_	5·10^8^ In sensitivity analysis median 10^6^ and deviation 10^2^	RNA copies/mL	[44]
Tracheo-bronchial + alveolar deposition, f_i_	0.3	–	[36]
Infective dose, D_50_	320 In sensitivity analysis 100–1000	RNA copies/mL	[4]
Decay time λ_IA_	0.6	1/h	[45]
Deposition (room) λ_D_	0.6	1/h	[46]

## 4 Results

### 4.1 Comparison of different spaces

#### 4.1.1 Air flow rates

**Fig. 6:**
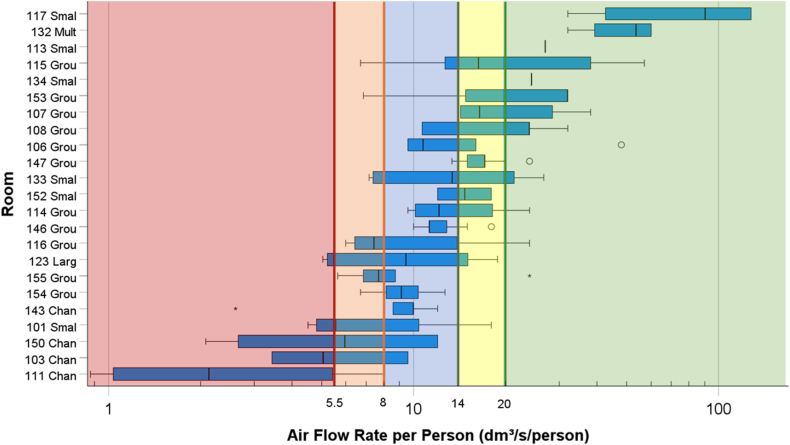
Boxplot showing hourly outdoor air flow rate per person in rooms. The boundaries of the box are Tukey's hinges. The median is identified by a line inside the box. The length of the box is the interquartile range (IQR) computed from Tukey's hinges [40]. Values greater than three IQRs from the end of a box are labelled as extreme, denoted with an asterisk (*) (IBM SPSS Statistic 28.0.1). Values greater than 1.5 but less than 3 IQRs from the end of the box are labelled as outliers (o). Colour codes: Red: Outdoor air flow rate <5.5 dm3/s/person; Orange: 5.5 < flow rate <8 dm3/s/person; Blue: 8 < flow rate <14 dm3/s/person; Yellow: 14 < flow rate <20 dm3/s/person; Green: Flow rate >20 dm3/s/person. Corresponding to the categories in EN16798–1:2019 [47] and recommendation for classrooms in ASHRAE 241–2023 [48]. (For interpretation of the references to colour in this figure legend, the reader is referred to the Web version of this article.)

The occupancy in the rooms varied during the day depending on their function. Sometimes the spaces were congested, e.g. changing rooms during arrival or departure, and dining rooms during lunch hour, whereas small group rooms were occupied by only a few people (Fig. 4). On the other hand, there were times when the spaces were unoccupied. Based on the reported occupancy in the room and the designed air flow rates, the outdoor air flow rates per person are shown in Fig. 6. The lowest air flow rates were in changing rooms showing minimum values down to less than 1 dm^3^/s/person and median values of less than 6 dm^3^/s/person. On the contrary, in several rooms air flow rates were more than 12 dm^3^/s/person.

#### 4.1.2 Airborne concentrations

**Fig. 7:**
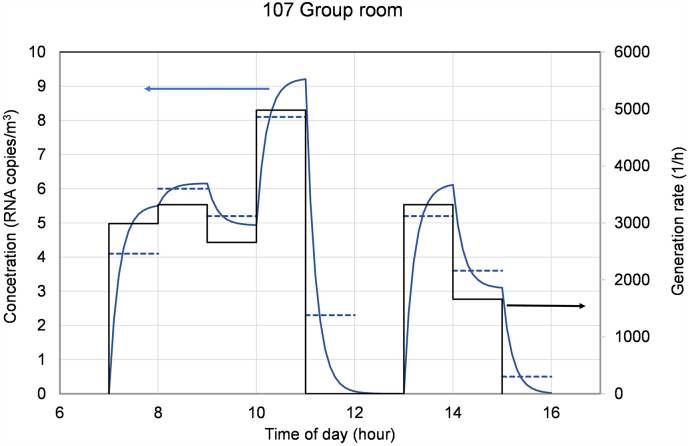
Changes in estimated pathogen generation rate and resulting concentration in an example room. Dashed lines represent the average concentration during each hour.

Besides the occupancy, the activity varied during the day in rooms and thereby influenced airborne pathogen emissions within the rooms. Based on hourly information on occupancy and activity in the room, the variation of the pathogen concentration was calculated for each room. An example of calculated airborne pathogen generation rates and resulting concentrations in one room of the day care centre is shown in Fig. 7.

**Fig. 8:**
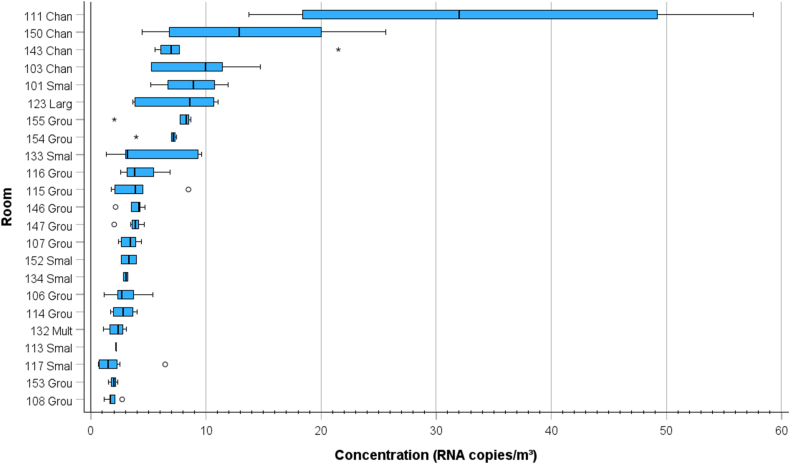
Boxplot of the estimated hourly average pathogen concentration in different rooms in the day care centre. Only times when there was occupancy in the room are included in the analysis. The median is identified by a line inside the box. The length of the box is the interquartile range (IQR) computed from Tukey's hinges [40]. Values greater than three IQRs from the end of a box are labelled as extreme, denoted with an asterisk (*). Values greater than 1.5 IQRs but less than 3 IQRs from the end of the box are labelled as outliers (o) (IBM SPSS Statistics 28.0.1).

The hourly average pathogen concentration for each room was calculated. The highest pathogen concentrations up to 58 RNA copies/m^3^ were estimated to occur in the changing rooms followed by the dining rooms (Fig. 8). At the other end there are rooms where the estimated concentration is low, only a few RNA copies/m^3^. Naturally, the concentration was dependent on occupancy, activity, and the air flow rate in the room.

**Fig. 9:**
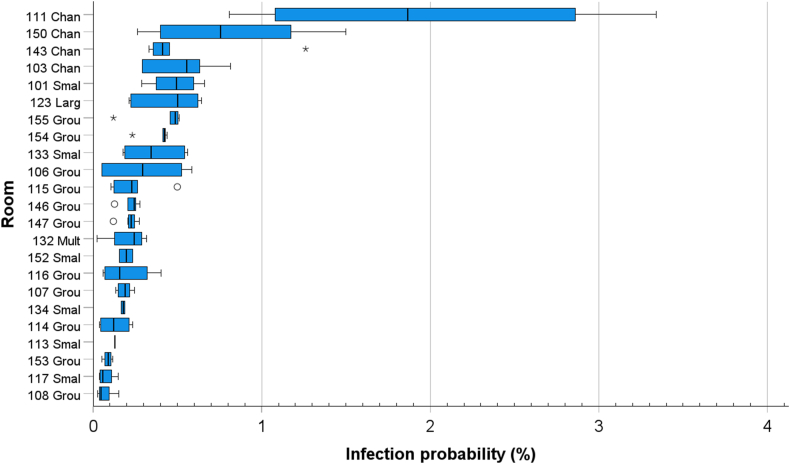
Boxplot of the variation in hourly infection probability at risk in each room. The median is identified by a line inside the box. The length of the box is the interquartile range (IQR) computed from Tukey's hinges [40]. Values greater than three IQRs from the end of a box are labelled as extreme, denoted with an asterisk (*). Values greater than 1.5 IQRs but less than 3 IQRs from the end of the box are labelled as outliers (o) (IBM SPSS Statistics 28.0.1).

Fig. 9 shows that there are large variations in the calculated probability of SARS-CoV-2 transmission between the rooms. The highest probability (3.3 %) was more than 100 times higher than the lowest one (0.03 %). The lowest probability is in the rooms characterised with high ventilation rates, a small number of persons and low-level activities (resting). In some rooms the statistically calculated generation rate of pathogens was low because of the small number of people in the room: the probability that an index person is in the room is directly proportional to the number of people in the room. The highest calculated risk is in the most occupied location, namely in the changing rooms (111, 143, 103 and 150) and in the rooms where lunch is served (101, 123 and 133). Despite reasonably high air change rates, the number of people in the space is high, so the flow rates per person are low.

**Fig. 10:**
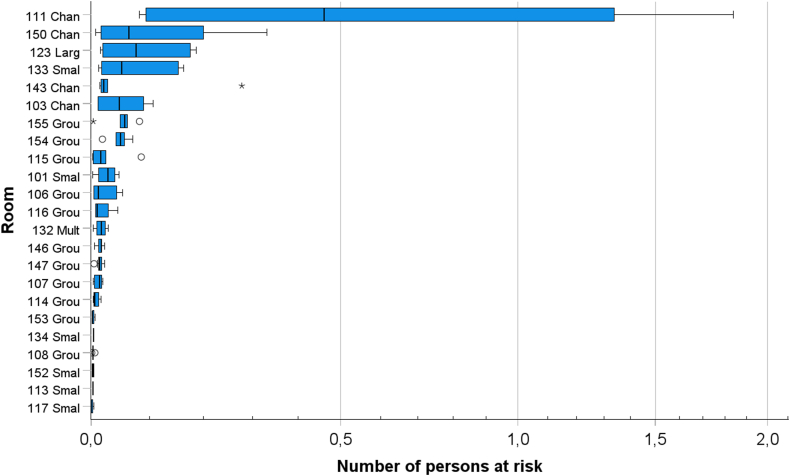
Boxplot of the variation of the hourly number of persons at risk in each room. The predicted number of persons at risk are for 1 h of exposure time and only for times when there was occupancy in the room. The median is identified by a line inside the box. The length of the box is the interquartile range (IQR) computed from Tukey's hinges [40]. Values greater than three IQRs from the end of a box are labelled as extreme, denoted with an asterisk (*). Values greater than 1.5 IQRs but less than 3 IQRs from the end of the box are labelled as outliers (o) (IBM SPSS Statistics 28.0.1).

The number of people at risk of transmission depends on the calculated infection probability in the room and the number of persons present in the room. The frequency of persons at risk calculated on hourly basis in all rooms in the day care centre is presented in Fig. 10. Most of the time, the estimated number of people at risk is very low. However, in some situations the estimated number of persons at risk is quite high. The highest number of persons at risk was in the changing room 111. Estimated daily secondary attack rate for the whole day care centre was 6.0 % based on the estimated number of persons at transmission risk divided by the number of total persons at the whole day care centre.

### 4.2 Sensitivity analysis

**Fig. 11:**
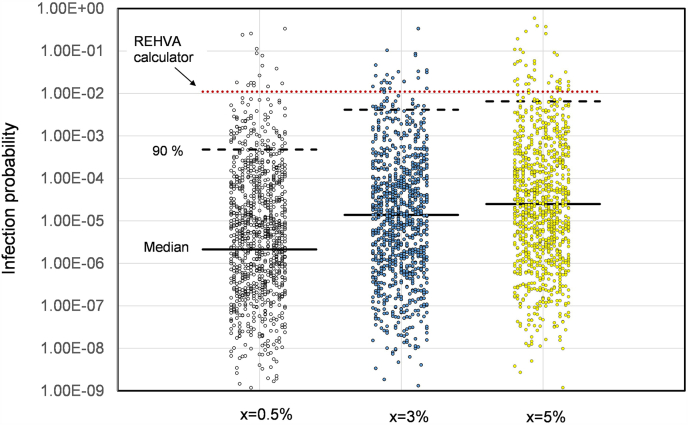
Monte Carlo simulations of infection probability in group room 116 for a 1-h period with three different disease prevalence. The median value of the simulations is shown by solid horizontal line while the 90th percentile is shown by dashed lines. The REHVA calculator results are shown for a quanta emission rate of 9 1/h.

The presented results were obtained using the parameter values shown in Table 3. Although these values were based on reasonable estimates found in the literature, it is well known that there are large uncertainties related to the emission of pathogens as well the number of inhaled viral copies that will cause infection (infectious dose). Therefore, the effect of varying the key parameters was studied using the Monte Carlo method by defining range of possible variable values and probability distributions. The variables were assumed to be normally distributed around their average values except for viral load which was modelled using log-normal distribution with a median value of 1.1x10^6^ RNA copies/mL and log standard deviation of 1.8 taken from Yang's research [25]. The calculations were made for a representative space, namely a home base (room 116), for 1 h of exposure. The simulations were made using a random number generator to sample values from the specified probability distributions for each variable. These calculations were repeated for several thousand times to get a median value for the infection probability. An example of the simulation results with different disease prevalence (0.5, 3 and 5 %) is presented in Fig. 11. For comparison, the REHVA calculator was used to calculate the infection probability in the same room for 1 h of exposure time [49]. As can be seen, the infection probability varies stochastically over a very wide range, with the difference between the median and the 90th percentile being about two orders of magnitude. The REHVA calculator gives values which are close to the simulated 90th percentiles with high disease prevalence.

**Fig. 12:**
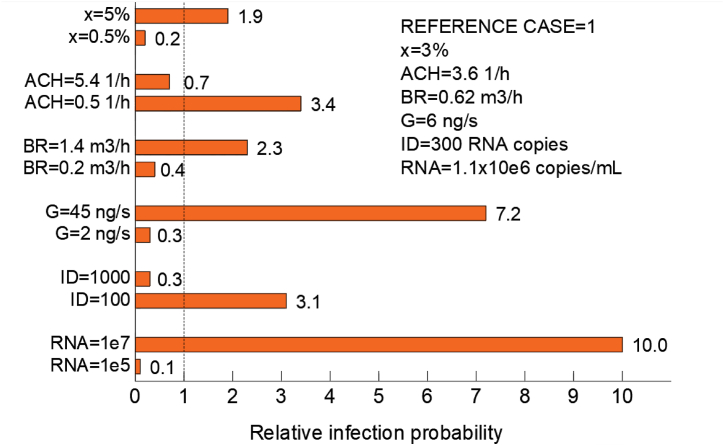
Median values for the relative risk of infection in cases where only one variable has been varied while the others remained constant in comparison to the reference case.

In the sensitivity analysis made for one representative room in the day care centre the values of different variables were varied one at a time to see how it affected the median infection probability. The most important variables to consider were found to be viral load in the respiratory tract and droplet emission rate (Fig. 12). The infectious dose leading to secondary transmission, ventilation rate and prevalence of the disease were also significant.

## 5 Discussion

The predictions show that there were large differences in the median infection probability between different spaces/rooms, and within a specific room the probability was dependent on time. The highest infection probability was observed in the hallways where children dress and undress before and after outdoor activities. These spaces are relatively small and crowded, and in peak hours the ventilation is insufficient in relation to the activities (rooms 103, 111, 143, 150). Also, in dining areas (rooms 101, 123, 133) and two of the group rooms (rooms 106, 155), the infection risk was ranked highest within this building. The airborne infection probability was intermediate in most of the group rooms and low in a small part of the group rooms where the spaces are not so crowded and/or the activity level was reported to be low. For example, in some group rooms (rooms 108 and 153) there were also quiet moments when the smallest children took a nap and the older ones rested. During these hours the pathogen emission rates and subsequently the airborne concentrations were low. The smallest predicted probability was in the small group rooms where there were only few persons at a time (room113 and 117). For such low occupancy, it is much less probable that there are infective persons than in rooms with higher occupancy.

The total daily infection probability of an individual person is the sum of the hourly probabilities, taking into consideration that there are also regular outdoor activities where the airborne infection risk can be assumed to be negligible [50]. Based on the daily schedule (8-h median) and the chosen initial values, the predicted average individual probability is about 1.7 % (IQR 0.5 %–3 %). This is proportional to the number of infective people, which depends on the prevalence of the disease in question in the community, in this case COVID-19. The estimated secondary attack rate based on the occupancy and estimated number of persons at risk for the whole day in our study was 6.0 %. This is in good agreement also with the reported secondary attack rates in day care centres of 1.2 %–9.6 % during the early phases of COVID-19 pandemic [51,52].

The simulations assumed that the pathogen concentrations were evenly distributed in each room. This is a common assumption when long-range aerosol transmission is modelled. However, this well-mixed approximation is often criticized because it does not take into account the spatial variation of the infectious particles [[53], [54], [55], [56]] resulting in underestimation of infection risk [57]. It is likely that the infectious particles are unevenly distributed especially in large spaces and the concentration as well as the secondary attack rate is highest near the stationary index case e.g. in restaurants and in public transport vehicles [58,59]. Several modifications to the Wells-Riley model have been introduced to consider the spatial distribution of the infectious particles [60,61].

In the design of the ventilation air flow rates for mixing ventilation, the deviation from the complete mixing assumption is adjusted with a term describing the ventilation effectiveness. Different definitions are given to point source and distributed source ventilation effectiveness [62]. Distributed source ventilation effectiveness definition is used e.g. in the EN 16798–3:2017 standard [63]. In the case of complete mixing the distributed source ventilation effectiveness is equal to 1. When the rooms are quite small (<50 m^2^) like in this study, the pathogen source is not stationary and the ventilation air flow rates are reasonably high it can be assumed that the ventilation effectiveness is close to 1, i.e. the well-mixed approximation is justified [62]. In addition, in a day care centre persons (especially children) do not stay still. They rather move around the room while playing and it can be assumed that also the release of pathogens (from an index case) in the day care centre is not a stationary point source. The other persons (susceptible) are also moving around the room and therefore they are exposed to the mean concentration of the room. Thus, it seems to be justified to assume that a well-mixed model produced acceptable estimations of relative risks between the rooms and that this information could be used for ranking of the spaces where the airborne infection mitigation measures are needed most. Such measures can include the use of face masks, limiting the number of people in crowded spaces, maintaining social distance, and the utilisation of portable air cleaners.

The calculated airborne virus concentrations varied between 1 and 58 RNA copies/m^3^. A few studies, mainly focusing on hospital premises, report measured airborne SARS-CoV-2 concentrations in indoor environments. In these studies, a very large variability in the concentrations with the known presence of infectious person was found, ranging from <1 to up to 94 000 RNA copies/m^3^, with the range most commonly being between 1000 and 3000 RNA copies/m^3^, with a few studies reporting only 1–50 RNA copies/m^3^ [[64], [65], [66], [67], [68], [69], [70], [71]]. The pathogen concentrations estimated in this study are at the lower end of the concentration reported. In the above-mentioned studies, there has been at least one index person in the room while the measurements were being taken. In this study, we utilised the prevalence of the disease (3 %) in estimating the number of index persons in the room which usually reduced the probable number of emitters and thus the emission rate of the pathogen.

The occupancy varied during the operating hours in a certain room from no to maximum number of occupants. The persons were located different rooms in different times and some of them were outdoors. Occasionally some rooms were extremely crowded and the estimated floor area per person was less than 1 m^2^/person. Similarly, the air flow rates per person varied strongly from less than 1 dm^3^/s/person up to 128 dm^3^/s/person. The median air flow rate was 12 dm^3^/s/person. The requirement for outdoor air flow rates in non-residential buildings depends on the floor area of the room and the number of occupants [47]. About 68 % of the rooms complied with the strictest air flow requirements (category I), which is applied to premises with children.

Although the relative risk can be modelled and predicted realistically, the absolute risk is almost impossible to define accurately. This is due to uncertainties and high variation in the key parameters, namely the pathogen emission rate and infectiousness of the virus. The inhaled dose of viruses causing infection is not well known. Moreover, the infective dose is highly variable and varies from person to person depending on the health and vaccination status, and prior infection [72]. SARS-CoV-2 variants are constantly evolving, and their prevalence has varied constantly over time and location. As the SARS-CoV-2 virus has new variants, its infectiousness has also changed. In the quanta-based infection risk models, the increased infectiousness needs to be taken into consideration by increasing the quanta generation rate, although the generation of droplets still remains the same.

The shedding rate of virions from the infected host is assumed to be proportional to the viral concentration of respiratory fluids, which can vary greatly between individuals. In an extensive study by Jones et al. [44] the log10 value of RNA copies in nasopharyngeal samples varied between 4 and 10, the mean being about 7 for presymptomatic, asymptomatic and mildly symptomatic subjects. The patients with Alpha and Delta variants were found to have ten times higher loads than with previous variants [44,72]. Similar distributions have been found in saliva samples where concentrations have ranged between 10^3^ and 10^10^ RNA copies per mL [73,74]. Additionally, the viral concentration in the respiratory specimens varies during the course of the infection, being highest at the onset of symptoms and then decaying constantly [44,75]. Another factor affecting virus emissions is vaccination status and previous infection. While vaccines protect against symptomatic infection and severe illness, they also reduce the shedding of viruses from infected people. Woodbridge et al. [76] showed that vaccination reduced the viral load of the Omicron variant, but its effect faded rapidly. However, it is known that the Omicron variant will be transmitted more easily than previous variants [77]. Yuasa et al. [78] showed that there was no significant difference in RNA copy numbers in nasopharyngeal swabs between the Omicron and Delta cases. This finding suggests that the difference in viral load did not cause the higher transmissibility of the Omicron variant, but it was possibly due to lower infectious dose and other factors [[78], [79], [80], [81]]. All this means that it is almost impossible to accurately predict the viral particle emission rate by an infected host at the individual level.

Interestingly it has been found that there is no big difference in the viral load between children and adults [26,44]. Also, in an extensive German study that investigated the prevalence of disease in day care centres, the rate of infections in staff was found to be higher compared to that of children [82]. In their meta-analysis, Zhu et al. [83] found that children are very rarely index cases in household transmission clusters. They also found that the secondary attack rate in paediatric household contacts was lower than in adult contacts.

Naturally, there may be other contributing factors to the elevated incidence rate among adults, like multiple close contact with children, the smaller number of the SARS-CoV-2 angiotensin-converting enzyme 2 (ACE2) receptors in the respiratory tract in children, and higher rates of other coronavirus infections in children might protect against severe SARS-CoV-2 infection in children [84,85].

Viner et al. [86] performed a systematic review and meta-analysis with SARS-CoV-2 secondary infection and found that children and adolescents (<20 years) were less contagious (44 % lower odds of secondary infection) compared with adults. This was especially the case with children younger than 14 [86]. Lachassinne et al. [87] also found in their seroprevalence studies in French day care centres that the share of SARS-CoV-2 infections in young children was low. However, recent studies have shown that the continuation of the epidemic with new SARS-CoV-2 variants and shifts in preventive measures and vaccination changed the situation [[88], [89], [90], [91]]. In addition, it seems that transmission between family members seemed more important than transmission within day care centres [87,92].

Preschool-aged children get an average of 5–8 respiratory infections per year according to Finnish data [93]. Those infections last an average of 7–12 days, during which the child cannot be taken to day care. In this case, a parent or other close adult must stay at home with the child. This causes significant financial costs for both the family and society [93].

## 6 Conclusions

A relatively simple but scientifically-based model for the transmission of airborne infectious pathogens was proposed in this study. The model captures the main factors affecting the risk of transmission in different settings and can benefit the implementation of effective control measures. The general structure of our model enables versatile use and modifications as more accurate information about the pathogen becomes available.

The model was applied in a day care centre for estimating the transmission probability and the number of people at risk of transmission on an hourly basis in all spaces where children and their nurses were regularly. The exercise unveiled large differences in the median infection probabilities between rooms, and the time dependence of the probability within the room, although the Monte Carlo simulations showed large stochastic variability between runs. This is valuable information for dimensioning the adequate clean air delivery rate for portable air cleaners, for example. The identification of hot spots can be useful for implementing other non-pharmaceutical measures required to mitigate airborne pathogen transmission risks.

One limitation of the model is that it only covers the airborne transmission of the pathogens. It will not take into consideration droplet transmission in close contact and possible fomite transmission, which might also be significant routes of infection in day care environments. The model also assumes uniform concentrations within the room, which might be an unrealistic assumption when close to the index person. There are also limitations when it comes to diseases that affect different age groups differently. The dynamics of contagiousness for a specific disease may be better understood by conducting separate analyses for children and adults interacting in shared spaces, such as households, public transportation, and commercial and educational facilities. However, when conducting large-scale studies or public health assessments, separating the population into children and adults may not always be practical. Thus, group-level risk assessment may be more relevant to understanding the overall transmission dynamics in these environments.

As shown in this study, the large variation in the parameters makes it difficult to make accurate estimations of airborne exposure and infection probability. Although there are several uncertainties in the initial data used for estimating the probability and the number of persons at risk, the quantification of relative risks between the spaces using the approach presented in this study is justified because the situations between the spaces are comparable and enable the identification of hot spots in indoor environments. We argue that the method presented in this study allows for the quantitative assessment of the relative importance of different spaces and times in the quantification of the airborne infection risk.

## Ethics declaration

Review and/or approval by an ethics committee was not needed for this study because the study involves information from freely available sources and did not include any human experimentation.

## Funding

This work was supported by the 10.13039/501100014438Business Finland project E3 Excellence in Pandemic Response and Enterprise Solutions.

## Data availability statement

The data that support the findings of this study are available on request from the corresponding author, [I.K].

## CRediT authorship contribution statement

**Ilpo Kulmala:** Writing – original draft, Methodology, Investigation, Conceptualization. **Aimo Taipale:** Writing – review & editing, Conceptualization. **Enni Sanmark:** Writing – original draft, Project administration. **Natalia Lastovets:** Writing – original draft, Investigation. **Piia Sormunen:** Resources, Project administration. **Pekka Nuorti:** Writing – review & editing. **Sampo Saari:** Writing – original draft. **Anni Luoto:** Investigation. **Arto Säämänen:** Writing – original draft, Investigation, Formal analysis.

## Declaration of competing interest

The authors declare that they have no known competing financial interests or personal relationships that could have appeared to influence the work reported in this paper.
